# Gut microbiota composition in travellers is associated with faecal lipocalin-2, a mediator of gut inflammation

**DOI:** 10.3389/fcimb.2024.1387126

**Published:** 2024-04-26

**Authors:** Javier Gandasegui, Andrea Vergara, Pedro Fleitas, Elisa Rubio, Mariana Fernandez-Pittol, Cristian Aylagas, Míriam Alvarez, Noelia Zancada, Daniel Camprubí-Ferrer, Jordi Vila, José Muñoz, Paula Petrone, Climent Casals-Pascual

**Affiliations:** ^1^ Barcelona Institute for Global Health (ISGlobal), Barcelona, Spain; ^2^ Department of Clinical Microbiology, Biomedical Diagnostic Center (CDB), Hospital Clínic of Barcelona, University of Barcelona, Barcelona, Spain; ^3^ CIBER Enfermedades Infecciosas (CIBERINFEC), Instituto Salud Carlos III, Madrid, Spain; ^4^ Tropical Medicine and International Health Department, Hospital Clínic, Barcelona, Spain

**Keywords:** microbiome, travel, diarrhoea, lipocalin-2, inflammation, machine learning

## Abstract

**Introduction:**

We examined the gut microbiota of travellers returning from tropical areas with and without traveller’s diarrhoea (TD) and its association with faecal lipocalin-2 (LCN2) levels.

**Methods:**

Participants were recruited at the Hospital Clinic of Barcelona, Spain, and a single stool sample was collected from each individual to perform the diagnostic of the etiological agent causing gastrointestinal symptoms as well as to measure levels of faecal LCN2 as a biomarker of gut inflammation. We also characterised the composition of the gut microbiota by sequencing the region V3-V4 from the 16S rRNA gene, and assessed its relation with the clinical presentation of TD and LCN2 levels using a combination of conventional statistical tests and unsupervised machine learning approaches.

**Results:**

Among 61 participants, 45 had TD, with 40% having identifiable etiological agents. Surprisingly, LCN2 levels were similar across groups, suggesting gut inflammation occurs without clinical TD symptoms. Differential abundance (DA) testing highlighted a microbial profile tied to high LCN2 levels, marked by increased *Proteobacteria* and *Escherichia-Shigella*, and decreased *Firmicutes*, notably *Oscillospiraceae*. UMAP analysis confirmed this profile’s association, revealing distinct clusters based on LCN2 levels. The study underscores the discriminatory power of UMAP in capturing meaningful microbial patterns related to clinical variables. No relevant differences in the gut microbiota composition were found between travellers with or without TD.

**Discussion:**

The findings suggest a correlation between gut microbiome and LCN2 levels during travel, emphasising the need for further research to discern the nature of this relationship.

## 1 Introduction

The gut microbiota plays a major role in deterring colonisation by exogenous microorganisms (Ducarmon et al., 2019). Two main mechanisms are involved: direct interaction of the commensal microbiota with pathogenic bacteria, and indirect interaction through activation of host immunity by production of immunomodulatory molecules (Buffie and Pamer, 2013; Kamada et al., 2013; Leslie and Young, 2015; Riddle and Connor, 2016). Therefore, a disruption of the gut microbiota could increase the susceptibility to infection by enteric pathogens (Kampmann et al., 2016). In the context of travel, the association of traveller’s diarrhoea (TD) and changes in the gut microbial diversity and taxonomic composition remains poorly understood. Gut microbiota during TD has been commonly characterised by an increase in *Proteobacteria* and *Bacteroidetes*, and a decrease in *Firmicutes* (Leo et al., 2019; Walters et al., 2020; Boolchandani et al., 2022; Worby et al., 2023). Some studies did not find differences in microbial diversity between healthy travellers and those experiencing diarrhoea upon their return (Rasko, 2017; Leo et al., 2019; Walters et al., 2020; Boolchandani et al., 2022); either between samples collected before and after diarrhoea episodes during travels (Walters et al., 2020). A recent study found that taxonomic composition of healthy travellers significantly changed over time (Boolchandani et al., 2022), which can be attributed to various factors inherent in travelling, including exposure to different microbial environments, changes in dietary habits, alterations in sleeping patterns, stress or medication intake (Riddle and Connor, 2016).

Diarrhoea can be classified as inflammatory or non-inflammatory, which has relevant therapeutic implications (Huicho et al., 1996). Previous studies have evaluated the degree of inflammation during infectious diarrhoea by examining markers of intestinal inflammation, which have been proposed as indicators for treatable inflammatory diarrhoea (Huicho et al., 1996; Bouckenooghe et al., 2000; Greenberg et al., 2002). One marker of bowel inflammation is the lipocalin-2 (LCN2), also known as neutrophil gelatinase-associated lipocalin (NGAL). LCN2 is a bacterially induced 21-kD glycoprotein secreted by neutrophils, hepatocytes and renal tubular cells, and it is critical for host defence (Bachman et al., 2009). In the bowel, LCN2 has the ability to interfere with bacterial iron uptake through competition with the siderophore enterobactin, thus preventing the colonisation and proliferation of exogenous and potentially pathogenic bacteria (Raffatellu et al., 2009). The association between LCN2 levels, inflammatory bowel diseases and microbiota has been previously explored (Moschen et al., 2016; Qiu et al., 2021; Watzenboeck et al., 2021), but never in the context of diarrhoeal diseases.

Therefore, in this work, we characterised the gut microbiota composition from travellers returning from tropical areas with and without TD, as well as its relation to LCN2 levels as a marker for gut inflammation and dysbiosis. Besides, we employed a set of conventional statistical tools and integrated unsupervised machine learning (ML) approaches. Unsupervised ML methods, such as dimensionality reduction analysis, were instrumental in handling the complexity of the microbiota data. These ML techniques not only helped reduce dimensionality but also revealed hidden patterns in the dataset that might not be immediately apparent through traditional analyses. By leveraging unsupervised ML, we aimed to identify a specific microbial profile associated with diarrhoea and/or gut inflammation after travel. This innovative approach enhances our ability to capture relationships within the microbiota, and characterise potential microbial signatures linked to adverse gastrointestinal outcomes in travellers.

## 2 Materials and methods

### 2.1 Study population and sample collection

A total of 67 participants returning from international travel were recruited during post-travel consultation at Hospital Clinic, Barcelona, Spain from August to October 2017. The study was approved by the Ethical Committee HCB/2023/0163 at Hospital Clinic and written informed consent was obtained from all participants included. Faecal samples were collected from all participants and Bristol scale type was recorded. Stool was immediately stored at -80°C after being homogenised with sterile phosphate-buffered saline (PBS).

Participants were classified according to the presentation of gastrointestinal symptoms related to diarrhoea. Firstly, participants were classified into two groups (hereinafter two-groups-criteria): Group 1, with diarrhoea (Bristol scale >4 and frequent stools, TD); and Group 2, no diarrhoea with solid and infrequent stools (Bristol scale <4, No TD). Afterwards, we identified the etiological agent responsible for the TD (see section below) and we subsequently classified the participants into three groups (hereinafter three-groups-criteria): Group 1A, diarrhoea with confirmed gastrointestinal pathogen (Confirmed TD); Group 1B, diarrhoea without pathogen confirmation but with numerous soft to watery stools (Bristol scale >4, Probable TD); and Group 2, no diarrhoea with solid and infrequent stools (Bristol scale <4, No TD).

### 2.2 Stool processing

Faecal samples were processed by standard methods for the detection of bacterial and parasitic pathogens: Blood agar (Oxoid^®^, Thermo Fisher, Spain), MacConkey agar (Becton Dickinson^®^, Heidelberg, Germany), CCDA agar (Becton Dickinson^®^) for *Campylobacter*, SS agar (Becton Dickinson^®^) for *Shigella* and *Salmonella*, CIN agar (Becton Dickinson^®^) for *Yersinia*, and Rappaport-Vassiliadis Salmonella Enrichment Broth (Becton Dickinson^®^) for the recovery of *Salmonella* and a subculture on SS agar after 24 hours incubation. The identification of isolated bacteria was performed by matrix-assisted laser desorption/ionisation time-of-flight (MALDI-TOF) mass spectrometry (Bruker, Bremen, Germany) except for *Salmonella/Shigella*, which were serotyped by agglutination with commercial antisera (Bio-Rad^®^, Marnes-la-Coquette, France). The detection of virulence genes of diarrheagenic *E.coli* was performed using an in-house multiplex PCR targeting the CVD432 probe of enteroaggregative *E. coli* and the *lt* and *st* genes of enterotoxigenic *E. coli* (Aranda et al., 2004; Zboromyrska et al., 2014). Fresh faecal samples were concentrated following the merthiolate formalin ether method to observe parasites, and additionally stained using a modified Kinyoun acid-fast stain for the detection of *Cryptosporidium* spp. and *Cyclospora cayetanensis* (Prince, 2008). LCN2 concentration was quantified using a modified enzyme immunoassay coupled with chemiluminescence in PBS-homogenised stool samples (Architect, Abbot).

### 2.3 DNA extraction and Illumina MiSeq *16S rRNA* amplicon sequencing

The PureLink™ Microbiome DNA Purification kit (Invitrogen™, Carlsbad, MA, USA) was used according to manufacturer’s instructions. DNA concentration and quality were measured with a Qubit 3.0 Fluorometer (Life Technologies, Ledeberg, Belgium).

The region V3-V4 from the 16S rRNA gene (amplicon size expected ~460 bp) was selected, and we used the primer pair described in the MiSeq rRNA Amplicon Sequencing protocol. Briefly, each DNA sample was subjected to dual barcoded PCR, amplifying the V3-V4 region of the 16S rRNA gene using the KAPA HiFi HotStart DNA Polymerase (KAPA Biosystems Inc., Wilmington, MA, USA). PCR products were purified by the Agencourt AMPure XP reagent (Beckman Coulter), and quantified using the Qubit 3.0 Fluorometer. Dual indices were attached using Nextera XT Index Kit (Illumina, Inc.) followed by the corresponding PCR amplification programme. After a second cleanup round, amplicons were quantified using the Qubit 3.0 Fluorometer. The library was prepared by pooling all PCR samples in equimolar concentration. Sequencing was performed on an Illumina MiSeq™ platform (Illumina, Inc.) according to manufacturer’s specifications to generate paired-end reads of 300 base-length in each direction.

### 2.4 Sequence processing and quality control

Sequencing data was then processed using QIIME2 (Bolyen et al., 2019). Amplicon sequencing data was obtained as a single file per run and per participant and imported in the Casava 1.8 paired-end demultiplexed fastq format. Forward reads were truncated at 287 pb position, and reverse reads at 249 bp, and then merged. Quality control and feature table construction were done using DADA2 and following standard pipelines. Taxonomy was assigned using Silva v138.1 database trained on the V3-V4 16S rRNA region with a Naive Bayes classifier.

### 2.5 Characterisation of gut microbiota

Downstream microbiome data analysis was performed with R software using a combination of phyloseq (McMurdie and Holmes, 2013), picante (Kembel et al., 2010), vegan (Oksanen, 2011), microbiomeR (Gilmore et al., 2019), and tidyverse (Wickham et al., 2019) packages.

#### 2.5.1 Alpha and beta diversity

Samples were rarefied at 3000 sequencing depth. For alpha diversity, we calculated Shannon index, Chao1 and Phylogenetic diversity (PD) parameters and we assessed their relationship with the clinical groups (for both two-group-criteria and three-group-criteria) by ANOVA or Kruskal-Wallis test, depending on the Shapiro test result of the distribution of the diversity values. The association between the alpha diversity and the LCN2 levels was assessed by spearman correlation. P-values were corrected for multiple testing by the Bonferroni method. We carried out both weighted and unweighted UniFrac PCoA for beta diversity estimation, considering the clinical groups as well as the LCN2 levels.

#### 2.5.2 Differential abundance (DA) testing

DA was tested at phylum, family and genus level. We set up a detection rate and prevalence threshold values of 1% and 10%, respectively. To explore the association of the different bacterial communities, we used a set of DA testing methods as previously recommended (Nearing et al., 2022). We employed AMCOM-BC and ALDEx to evaluate the association with the clinical presentation of TD (described as a categorical variable); whereas we used the conventional spearman correlation, adjusted for multiple testing by the Bonferroni method, and ALDEx to assess the association between LCN2 (a continuous variable) and the microbiome composition. We established a significant level of p-value<0.05. These analyses were performed using the R packages ALDEx2 (Fernandes et al., 2013), ANCOM (Lin et al., 2022).

Afterwards, we used Uniform Manifold Approximation and Projection (UMAP) on the abundances of bacteria. UMAP is a dimensionality reduction technique used in machine learning and data analysis. It aims to represent high-dimensional data in a lower-dimensional space, preserving both global and local data structures. UMAP is particularly effective in revealing complex patterns and relationships in the data, making it useful for visualisation. UMAP visualisations were carried out for relative abundance of bacteria at phylum, family and genus level to explore the capacity of the bacterial communities to discriminate participants with low and high LCN2 levels, as well as participants with and without TD. We firstly normalised the bacteria’s relative abundance. Then, we assessed the capacity of different groups of bacteria attending to the results obtained by conventional DA testing: (i) using the abundance of all bacteria at the three taxonomic level, (ii) using those bacteria statistically significant by spearman correlation before adjusting for multiple testing by the Bonferroni method, (iii) using those bacteria statistically significant by spearman correlation after adjusting for multiple testing by the Bonferroni method, and (iii) using those bacteria communities significantly associated by either ALDEx or ANCOM-BC. These analyses were performed in R using the umap package (McInnes et al., 2018).

## 3 Results

### 3.1 Study subjects and faecal microbiological culture

We obtained complete microbiome data from 61 out of the 67 participants initially recruited (**Table 1**). Complete participant information can be found in the **Supplementary Table S1**. The travel destination was highly diverse within these participants: South America, the Caribbean, South-East Asia or Africa. The identified pathogens included Enteroaggregative *E. coli* (N=5, EAEC), enterotoxigenic *E. coli* (N=4, ETEC), ETEC+EAEC (N=2), *Giardia lamblia* (N=2), *Campylobacter jejuni* (N=2), *G. lamblia* + *C. jejuni* (N=1), *G. lamblia* + EAEC (N=1), and *Salmonella choleraesuis* (N=1). There were no significant differences between the groups in terms of age or sex. As expected, the criteria for defining TD, which gather Bristol scale and frequency of stools, differed between Group 1 and Group 2. Also, an overall tendency of higher levels of LCN2 in stool can be observed in participants with TD, particularly on those with microbiologically confirmed TD. However, LCN2 values were highly variable within our groups, resulting in non-significant differences.

**Table 1:** Characteristics of the participants included in the study after being divided into clinical groups attending to the two-group-criteria or the three-group-criteria.

Two-group-criteria				
	Group 1 - TD (N=45)	Group 2 - No TD (N=16)	p-value	
Age (years) (median, IQR)	33 (27-43)	33 (26-41)	0.784	
Women (%)	68.9	75	0.756	
Bristol scale (median, IQR)	6 (5-7)	2 (2-2.25)	<0.001	
N of stools (median, IQR)	6 (4-7)	3 (1-4)	0.039	
LCN2 (ng/mL) (median, IQR)	2919 (1352-6804)	2175.5 (794.2-9067.7)	0.908	
Three-group-criteria				
	Group 1A - Confirmed TD (N=18)	Group 1B - Probable TD (N=27)	Group 2 - No TD (N=16)	p-value
Age (years) (median, IQR)	28.5 (27-32.25)	39 (31.5-49)	33 (26-41)	0.064
Women (%)	83.3	59.2	75	0.229
Bristol scale (median, IQR)	6 (4-7)	6 (5-7)	2 (2-2.25)	<0.001
N of stools (median, IQR)	6 (4.75-8.5)	5 (3-6)	3 (1-4)	0.075
LCN2 (ng/mL) (median, IQR)	4166.5 (2676.7-8412.7)	2097 (408.5-6398.5)	2175.5 (794.2-9067.7)	0.255

N, number; IQR, Interquartile range; LCN2, faecal lipocalin-2.

### 3.2 Alpha and beta diversity

**Figure 1:**
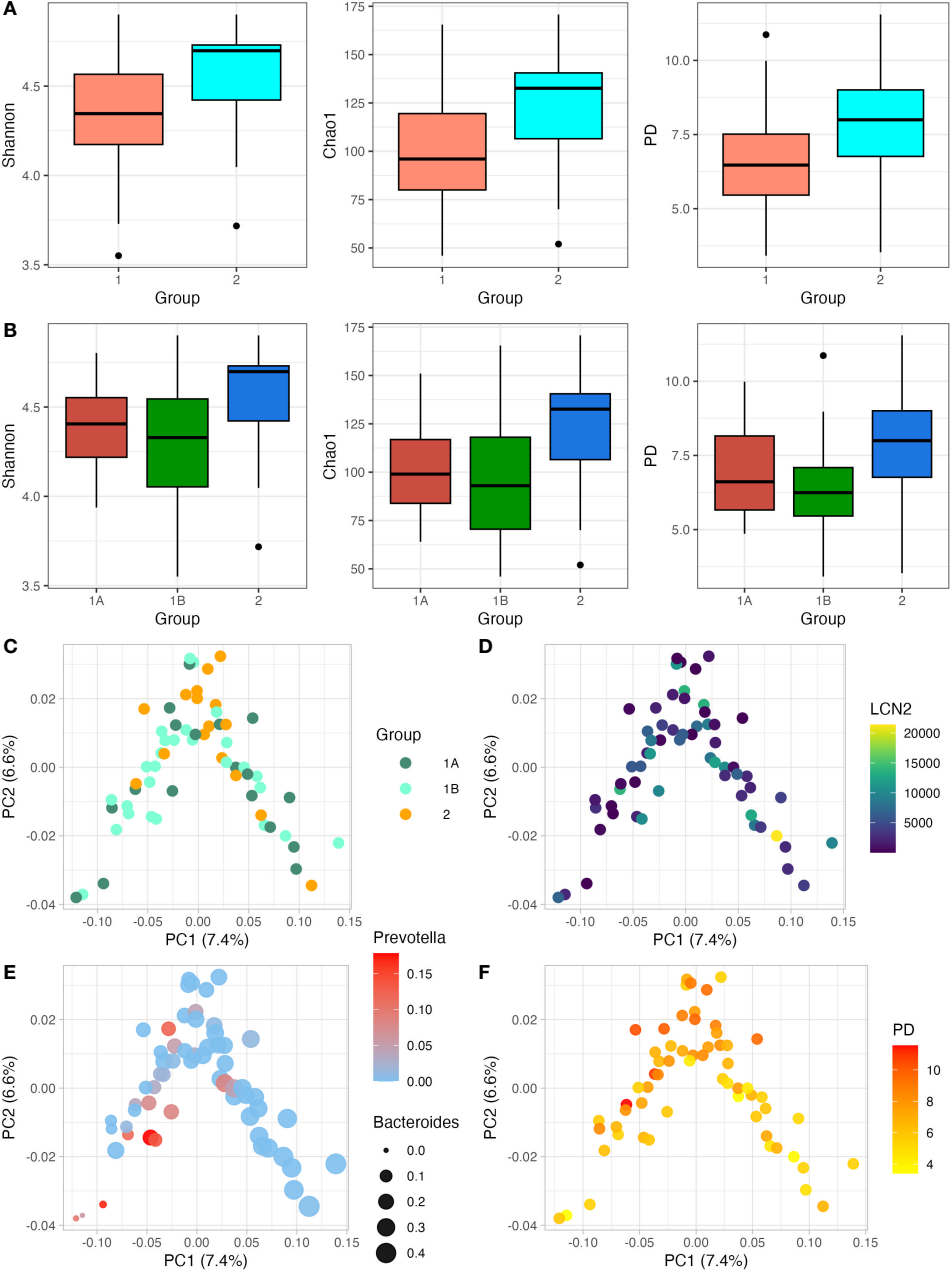
Relationship between alpha diversity and beta diversity with LCN2 levels and clinical presentation of TD. **(A, B)**; Shannon, Chao1 and Phylogenetic diversity (PD) index were estimated. In **(A)** participants are divided into two groups (TD and no TD; Group 1 and Group 2); whereas in **(B)** participants are divided into three groups (Confirmed TD, Probable TD and no TD; Group 1A, Group 1B and Group 2, respectively). **(C, D)** show the relation between the beta diversity weighted unifrac PCoA and the clinical presentation of TD **(C)** and LCN2 levels **(D)**; **(E, F)** show the relation of beta diversity weighted unifrac PCoA with the relative abundance of *Pevotella/Bacteroides* and alpha diversity expressed as PD, respectively. with weighted unifrac PCoA. Along with the y- and x-axis, the percentage of variance explained by PC1 and PC2 is indicated.

After data quality control and filtering, we got a mean number of sequences of 7148 (range 3147-11731). We observed that participants showing TD presented a lower number of sequences (6778) when compared to participants without TD (8189). Considering that during the library preparation all DNA samples were normalised before being pooled, this difference could be due to the higher proportion of human and/or fungal DNA in those watery and more frequent stools during diarrhoea episodes. Samples were rarefied at 3000 sequencing depth to keep all samples in dowstream analysis. Regarding the clinical groups, all alpha diversity estimators tended to be higher in the groups without diarrhoea (**Figures 1A, B**). After correcting by the Bonferroni method, p-values for Shannon, Chao1 and Faith’s PD for the two-group-criteria were 0.076, 0.020, 0.032, respectively. P-values for Shannon, Chao1 and PD for the three-groups-criteria were 0.156, 0.068, 0.060, respectively. Although an overall tendency of lower diversity in those participants with diarrhoea, all these diversity estimators become non-significant when three clinical groups were considered, which can be due to the similarity between the participants with confirmed-TD and probable-TD. Regarding the association between LCN2 levels and alpha diversity, the Spearman correlation showed a negative association in all cases, although it was not significant (p-value>0.05) (**Supplementary Figure S1**). Then, we selected only those participants that showed clinical diarrhoea, but the association between LCN2 levels and alpha diversity was still non-significant (p-value>0.05) (**Supplementary Figure S2**). We also carried out weighted unifrac PoCA for beta diversity analysis, considering the clinical groups as well as the LCN2 levels (**Figures 1C, D**). There were no patterns of clustering related to the two outcomes by PCoA in any case. Also, beta diversity shows certain structure: PC1 seems to be influenced by to the abundance of *Prevotella* and *Bacteroides* (**Figure 1E**), whereas PC2 shows relation with alpha diversity (**Figure 1F**).

### 3.3 Differential abundance testing

#### 3.3.1 Relation to faecal LCN2 levels

**Figure 2:**
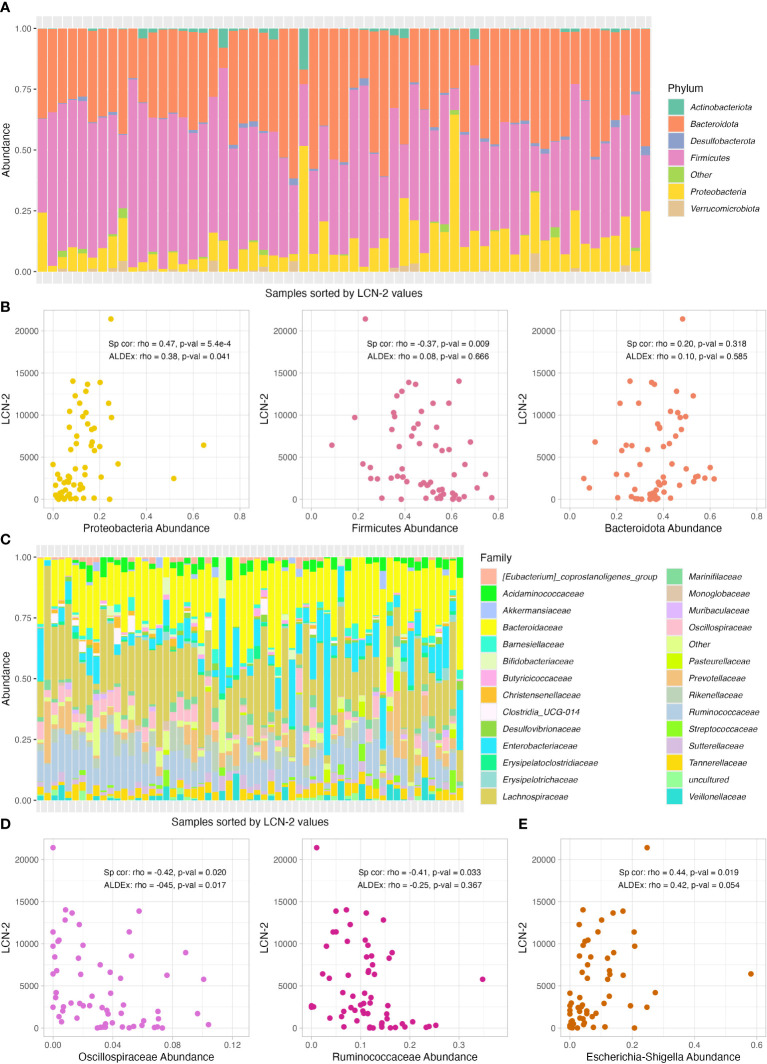
Relative abundance of bacterial phylum and correlation analysis with LCN2 levels. **(A)** Relative abundance of bacterial phyla per sample, which are shown sorted by faecal LCN2 levels (lower levels on the left and higher on the right). **(B)** Scatter plot of the correlation between *Bacteroidetes*, *Firmicutes* and *Proteobacteria* abundance and LCN2 levels. **(C)** Relative abundance of bacterial family per sample, which are shown sorted by faecal LCN2 levels (lower levels on the left and higher on the right). **(D, E)** Scatter plots of the correlation between *Oscillospiraceae*, *Ruminococcaceae* and *Escherichia-Shigella* abundance and LCN2 levels. Spearman (Sp cor) and ALDEx correlation coefficient (rho) and p-values are shown in each graph for each taxonomic group.

First, we explored the differences in the abundances of bacterial communities at phylum level. The three most common bacterial phyla in our samples were *Firmicutes*, *Bacteroidota* and *Proteobacteria* (**Figure 2A**). When comparing the correlation with faecal LCN2 levels by Bonferroni-corrected spearman correlation, we found a direct correlation between the relative abundance of *Proteobacteria* (rho=0.47, p-value=5.4e-4); as well as an inverse correlation between *Firmicutes* and LCN2 levels (rho=-0.37, p-value=0.009). No correlation was found when *Bacteroidota* was considered (p-value=0.318) (**Figure 2B**). When using ALDEx, only *Proteobacteria* phylum was still significant (rho=0.38; p-value=0.041) and *Firmicutes* was not significantly different (p-value=0.666) (**Figure 2B**). A total of 28 bacterial families were identified (**Figure 2C**) (**Supplementary Table S2**). Two families, *Oscillospiraceae* and *Ruminococcaceae*, exhibited an inverse spearman correlation with LCN2 levels, with p-values of 0.020 and 0.033, respectively (**Figure 2D**). Only *Oscillospiraceae* was significantly correlated with LCN2 by ALDEx (rho=-0.42, p-value=0.017). Finally, we identified a total of 56 bacterial genus (**Supplementary Table S3**). In this case, only *Escherichia-Shigella* abundance was directly correlated with LCN2 levels by spearman correlation (**Figure 2E**).

**Figure 3:**
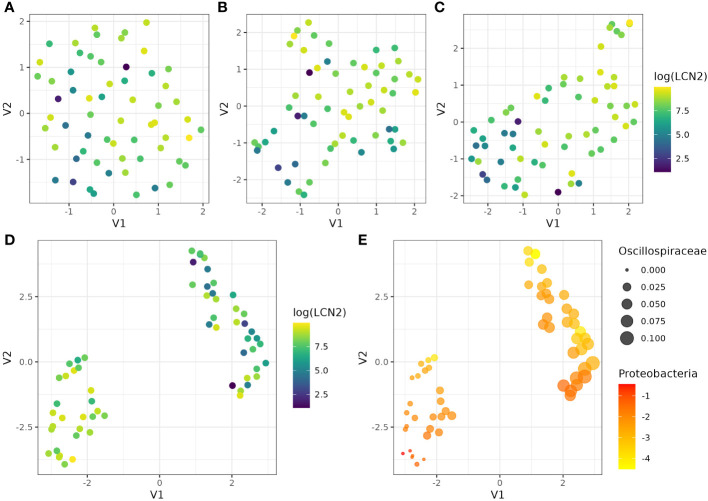
UMAP visualisation of cluster and LCN2 level patterns using the abundance of different bacterial communities. UMAP was performed using **(A)** the abundance of all bacteria, **(B)** those bacteria significantly associated with LCN2 by spearman correlation before adjusting the p-value by the Bonferroni method, **(B)** those bacteria significantly associated with LCN2 by spearman correlation after adjusting the p-value by the Bonferroni method, and **(C)** those bacteria significantly associated with LCN2 by ALDEx. **(E)** shows the UMAP results using those bacteria significantly associated with LCN2 by ALDEx, *Proteobacteria* and *Oscillospiraceae*. UMAP identifies two clusters with samples from similar bacterial spaces, represented in a lower-dimensional space. In **(A–D)**, we used log transformed levels of LCN2, which facilitate the visualisation of the differences between participants. In **(E)**, we represented *Oscillospiraceae* abundance by the size of the dots, whereas *Proteobacteria* log transformed abundance was represented by a scale colour from yellow (lower abundances) to red (higher abundances).

Then, we used UMAP to assess if the abundance of different groups of bacteria communities were able to discriminate between participants with lower and higher levels of LCN2 as a proxy for gut inflammation. First, we evaluated all the abundance at the three taxonomic levels (**Figure 3A**), with no evidence of clustering. Similar results were observed when we used the abundances of bacteria that were significantly associated by spearman correlation before adjusting the p-value by the Bonferroni method (**Figure 3B**). After adjusting the spearman correlation for multiple testing, UMAP was able to distinguish between participants with different levels of LCN2 (**Figure 3C**); however, we obtain clear evidence of two groups of participants with different LCN2 levels when we used in UMAP those bacteria abundances that were statistically associated only by ALDEx (**Figure 3D**), which are *Proteobacteria* and *Oscillospiraceae* (**Figure 3E**). Thus, we confirmed that the specific microbial profile, particularly determined by an increase in *Proteobacteria* phylum and a decrease in *Oscillospiraceae* family, is correlated with faecal high levels of LCN2 and gut inflammation. Nevertheless, it is important to interpret these results cautiously. While the correlation between the gut microbiota and LCN-2 levels post-travel is apparent, the absence of longitudinal or concomitant diseases data impedes our ability to conclusively determine whether bowel inflammation is a cause or consequence of the altered gut microbiome composition resulting from travel. The different groups of bacteria included in this analysis can be extracted from the **Supplementary Tables S2**, **S3**.

#### 3.3.2 Relation to the clinical presentation of TD

We performed DA testing between participants with clinical diarrhoea and without it. ANCOM-BC and ALDEx were used as conventional DA testing tools. At phylum, family and genus level, neither ANCOM-BC and ALDEx showed any significant differences (p-values>0.05), particularly for those families that were associated with LCN2 (**Supplementary Figure S3**), which reinforces the lack of association between gut inflammation and TD. As we were not able to identify any bacteria community related to TD, we did not continue with UMAP to assess potential bacterial profiles associated with it.

## 4 Discussion

Here, we show the results of the relationship between the gut microbiota composition in individuals returning from tropical areas, and its association with gut inflammation expressed as LCN2 concentration. We obtained microbiome data from 61 travellers, out of which 45 presented TD. We identified the etiological agent of the diarrhoea using conventional microbiological methods in 18 out of 45 participants (40%), which is consistent with previous reports (Centers for Disease Control and Prevention (CDC), 2019). Contrary to what would be expected, LCN2 levels were similar across the two or three study groups, particularly between those patients showing acute TD with confirmed pathogen and those individuals without TD. This suggests that travellers without clinical symptoms of diarrhoea might show some degree of gut inflammation after travel. As commented before, the travel itself significantly alters the composition of the gut microbiota (Youmans et al., 2015; Boolchandani et al., 2022); thus, we would speculate that the sole colonisation of the gut by exogenous bacteria may produce some degree of immune response and increase LCN2 levels, as previously observed in nasopharyngeal mucosa (Bachman et al., 2009).

We also assessed a set of diversity parameters and its relationship with the clinical presentation of TD as well as against LCN2 concentration. Alpha diversity was lower in participants presenting TD than those that did not show diarrhoea after travel. Diarrhoea has been reported to produce a marked reduction in taxonomic richness and diversity compared to age-matched and location-matched healthy individuals (Pop et al., 2014; The et al., 2018). We did not observe such marked reduction in diversity probably due to the extreme heterogeneity in etiological agents, place of origin, and individuals’ characteristics with TD. Moreover, TD gathers a highly divergent group of clinical presentations, from self-limiting diarrhoea to dysentery. We could thus expect that, although resulting in TD, the alteration in microbiome diversity strongly varies from one patient to another. No correlation was either found between LCN2 levels and either alpha or beta diversity. Previous studies reported a reduction in species diversity when LCN2 concentration was high (Moschen et al., 2016); however, this was not observed in our data.

Then, we assessed the relation between bacterial phylum, family and genus composition, and LCN2 concentration using a set of conventional DA testing tools followed by an unsupervised ML method to validate this association with specific microbial communities. The lack of consistency across the different tools for DA testing, suggesting that researchers should use a consensus approach based on multiple differential abundance methods to help ensure robust biological interpretations, has been recently highlighted (Nearing et al., 2022). In this context, ANCOM-BC and ALDEx have been described as conservative and reproducible methods for DA testing (Fernandes et al., 2013; Lin and Peddada, 2020), and they have been recommended as tools less prone to produce false positive associations (Nearing et al., 2022).

As a complementary analysis, we also assessed the discriminatory capacity of the DA tests between participants with lower and higher LCN2 levels using UMAP. This method efficiently reduced the dimensionality of significant bacterial communities, particularly ALDEx-derived bacterial abundances, allowing for the identification of distinct clusters that group patients with similar bacterial profiles. Notably, when applied to these abundances, UMAP reveals two clusters with significantly different levels of LCN2, highlighting its discriminatory power in capturing meaningful patterns associated with specific clinical variables. This underscores UMAP’s efficacy in visualising complex microbial data and its potential to contribute valuable insights into the relationships between bacterial profiles and clinical parameters such as LCN2 levels. In addition, we observed the importance of adjusting conventional statistical tests for multiple testing, particularly using conservative approaches such as the Bonferroni method, which is not always done in microbiome studies. UMAP has been previously used as a dimensionality reduction method for beta diversity microbiome research (Armstrong et al., 2021); however, we applied UMAP on abundances of bacteria. Our results have shown the utility of this tool in assessing DA testing approaches and its utility can expand to the confirmation of the different microbiota profiles obtained by more than one DA analyses, as previously recommended (Nearing et al., 2022).

Using the combination of the conventional spearman correlation, ALDEx and UMAP, we identified specific microbial communities associated with faecal LCN2 and gut inflammation. This profile showed an increase in *Proteobacteria* phylum, with a major contribution of the genus *Escherichia-Shigella*; and a decrease in *Firmicutes* phylum, particularly the families *Ruminococcaceae* and more significantly, *Oscillospiraceae*. This profile was not only associated with TD in the majority of the previous studies (Leo et al., 2019; Walters et al., 2020; Boolchandani et al., 2022), but also in other works using animal models to study other inflammatory bowel diseases (Moschen et al., 2016; Qiu et al., 2021). These findings are also consistent with a previous study, which found that LCN2 deficiency resulted in an increase of the bacterial genus *Angelakisella*, belonging to the family *Ruminococcaceae* (Qiu et al., 2021); and this family has shown to be health-promoting bacteria (Biddle et al., 2013). Also, previous studies have reported members of the family *Oscillospiraceae* have several positive benefits in human health (Konikoff and Gophna, 2016; Yang et al., 2021), and the presence of the genus *Oscillospira* is reduced in diseases that involve inflammation (Gophna et al., 2017). In this study, these two families may also be protective against gut inflammation. The genus *Escherichia-Shigella*, as observed in this work, is among the most commonly overgrown bacteria in many conditions involving inflammation; this has been observed in other Enterobacterales in conditions such as stroke, inflammatory bowel disease, colorectal cancer, or antibiotic treatment (Zeng et al., 2017; Díaz-Marugan et al., 2023). However, whether this phenomenon triggers or is a consequence of inflammation remains unclear (Manichanh et al., 2012). This profile should be further evaluated, preferably in longitudinal studies, to clarify whether LCN2 concentration and bowel inflammation is cause or consequence of the alteration of the gut microbiome composition. By contrast, none of these bacterial communities were associated with having diarrhoea, which supports the intrinsic inflammatory response in the gut after travel regardless of the clinical presentation of the TD.

One limitation of this work relies on using only microbiological methods to detect the etiological agent responsible for diarrhoea. Molecular methods, such as multiplex PCR-based approaches, could have increased the detection rate, particularly for those patients infected by viruses (Ko et al., 2006). However, our detection rate was higher than that reported in other studies using PCR-based methods (Boolchandani et al., 2022), so we believe, in this particular study, that the use of molecular methods might have had a marginal increase in the detection of pathogens. Also, we partially addressed this limitation by splitting the study population in groups based on etiological certainty (TD *vs*. Confirmed and Probable TD). A second limitation could be the small sample size, particularly in the context of TD. As commented above, TD gathers multiple etiological agents and clinical manifestations, and in our particular case, an heterogeneous group of participants. As a potential consequence of these limitations, we were unable to find statistical associations between individuals with or without TD, and the microbiome composition.

In conclusion, our work indicates a correlation between gut microbiome composition and LCN2 concentration in the context of travelling. Although the clinical definition of TD has shown to be challenging, we observed a specific microbial profile associated with gut inflammation. Further studies must clarify whether this particular microbiome signature is a consequence or induces gut inflammation not only in patients with TD, but also in other inflammatory bowel conditions. These studies will provide insights into potential interventions aimed at preventing or ameliorating these conditions.

## Data availability statement

All statistical results were available in supplementary tables. The microbiome data used in this study can be found at Sequence Read Archive: PRJNA1068937. Code required to reanalyze the data reported in this paper is available on Github: https://github.com/Gandasegui/microbiome_TD. Any additional information required to reanalyze the data reported in this paper is available from the corresponding authors upon request.

## Ethics statement

The studies involving humans were approved by Ethical Committee HCB/2023/0163 at Hospital Clinic of Barcelona. The studies were conducted in accordance with the local legislation and institutional requirements. The participants provided their written informed consent to participate in this study.

## Author contributions

JG: Data curation, Formal analysis, Investigation, Methodology, Visualization, Writing – original draft, Writing – review & editing, Validation, Resources. AV: Conceptualization, Investigation, Methodology, Resources, Writing – original draft, Writing – review & editing. PF: Formal analysis, Investigation, Methodology, Writing – original draft, Writing – review & editing. ER: Conceptualization, Investigation, Methodology, Writing – review & editing. MF-P: Investigation, Writing – review & editing. CA: Investigation, Resources, Writing – review & editing. MA: Investigation, Resources, Writing – review & editing. NZ: Investigation, Writing – review & editing. DC-F: Investigation, Writing – review & editing. JV: Conceptualization, Investigation, Resources, Writing – review & editing. JM: Investigation, Writing – review & editing. PP: Investigation, Methodology, Supervision, Visualization, Writing – review & editing. CC-P: Conceptualization, Data curation, Formal analysis, Funding acquisition, Investigation, Methodology, Resources, Supervision, Writing – original draft, Writing – review & editing.

## Supplementary material

Supplementary Table S1 Participant and sample metadata.

Supplementary Table S2 Bacteria communities at family level and its statistical association with LCN2 levels by spearman correlation and ALDEx.

Supplementary Table S3 Bacteria communities at genus level and its statistical association with LCN2 levels by spearman correlation and ALDEx.
