# Supplementary material for: Gut microbiota composition in travellers is associated with faecal lipocalin-2, a mediator of gut inflammation
Source: Front Cell Infect Microbiol. 2024 Apr 26;14:1387126. doi: 10.3389/fcimb.2024.1387126 (PMC11082338; doi:10.3389/fcimb.2024.1387126)
Supplement: Supplementary file 1 [file DataSheet_1.docx]

**Supplementary Figures S1 to S3**


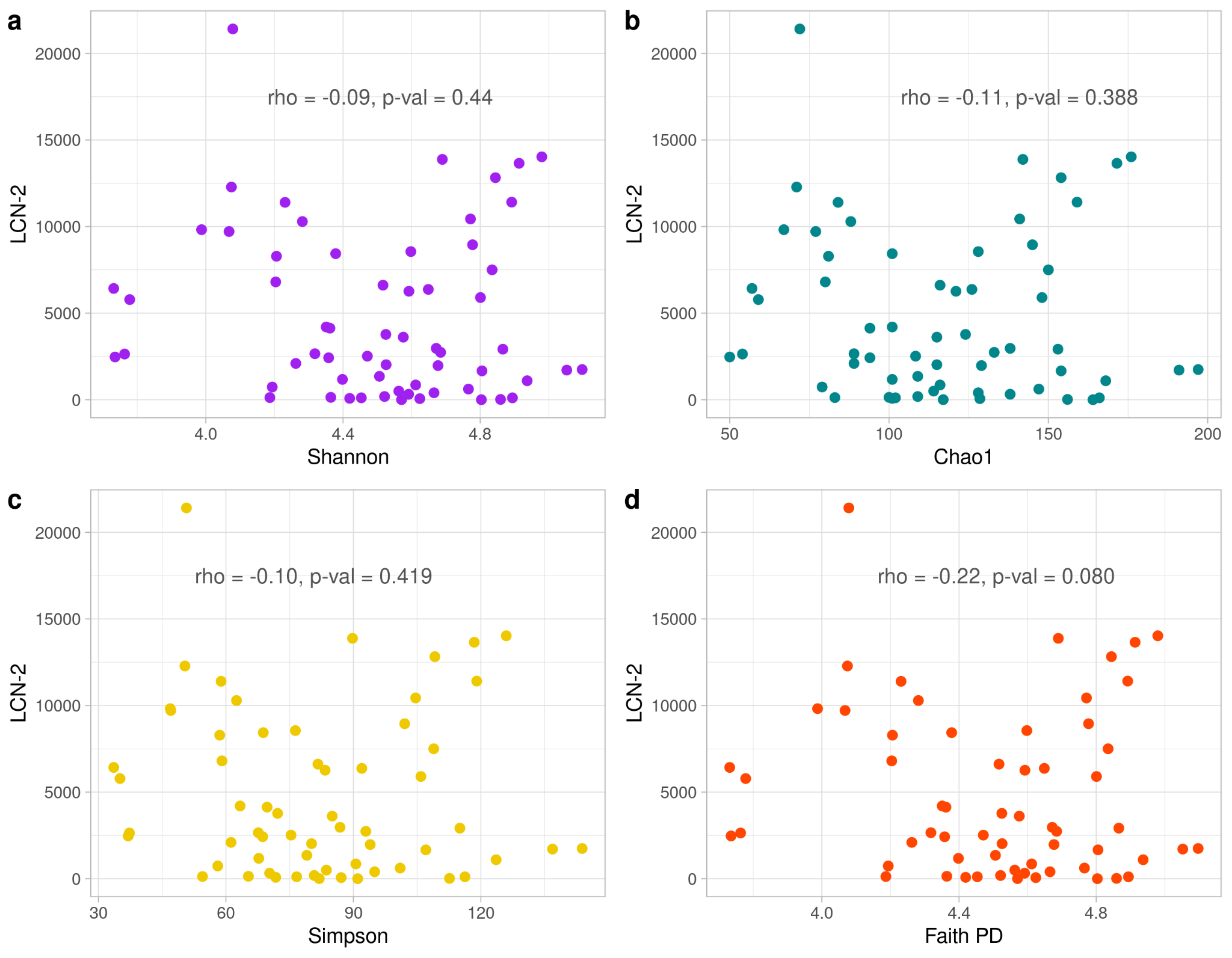


**Figure S1. Correlation between alpha diversity and LCN2 levels only in all patients TD.** We estimated Shannon (a), Chao1 (b), Simpson (c), and Phylogenetic diversity (PD) (d). Spearman correlation coefficient and p-values are shown in each graph for each alpha diversity estimator.


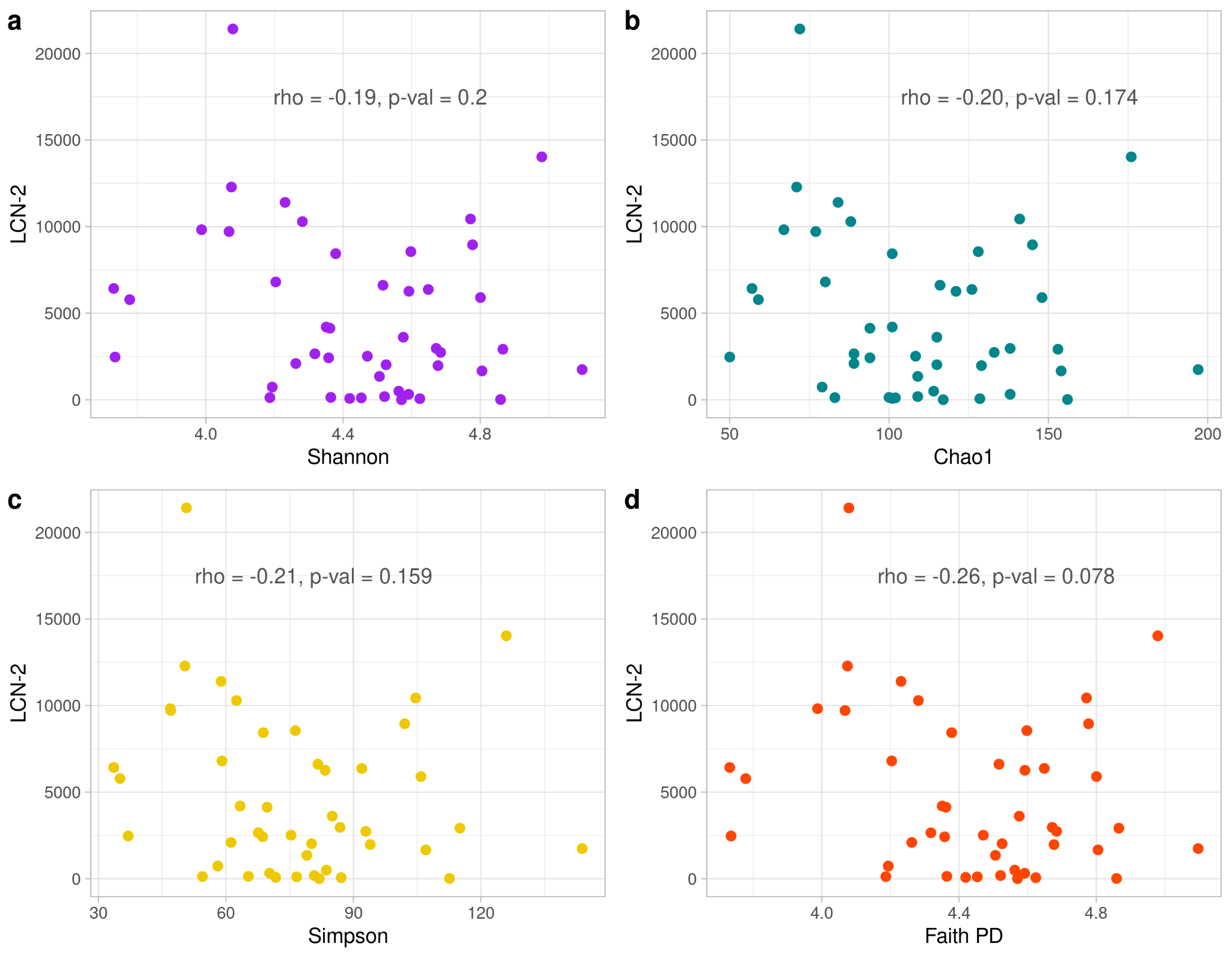


**Figure S2. Correlation between alpha diversity and LCN2 levels only in patients showing active clinical TD.** We estimated Shannon (a), Chao1 (b), Simpson (c), and Phylogenetic diversity (PD) (d). Spearman correlation coefficient and p-values are shown in each graph for each alpha diversity estimator.


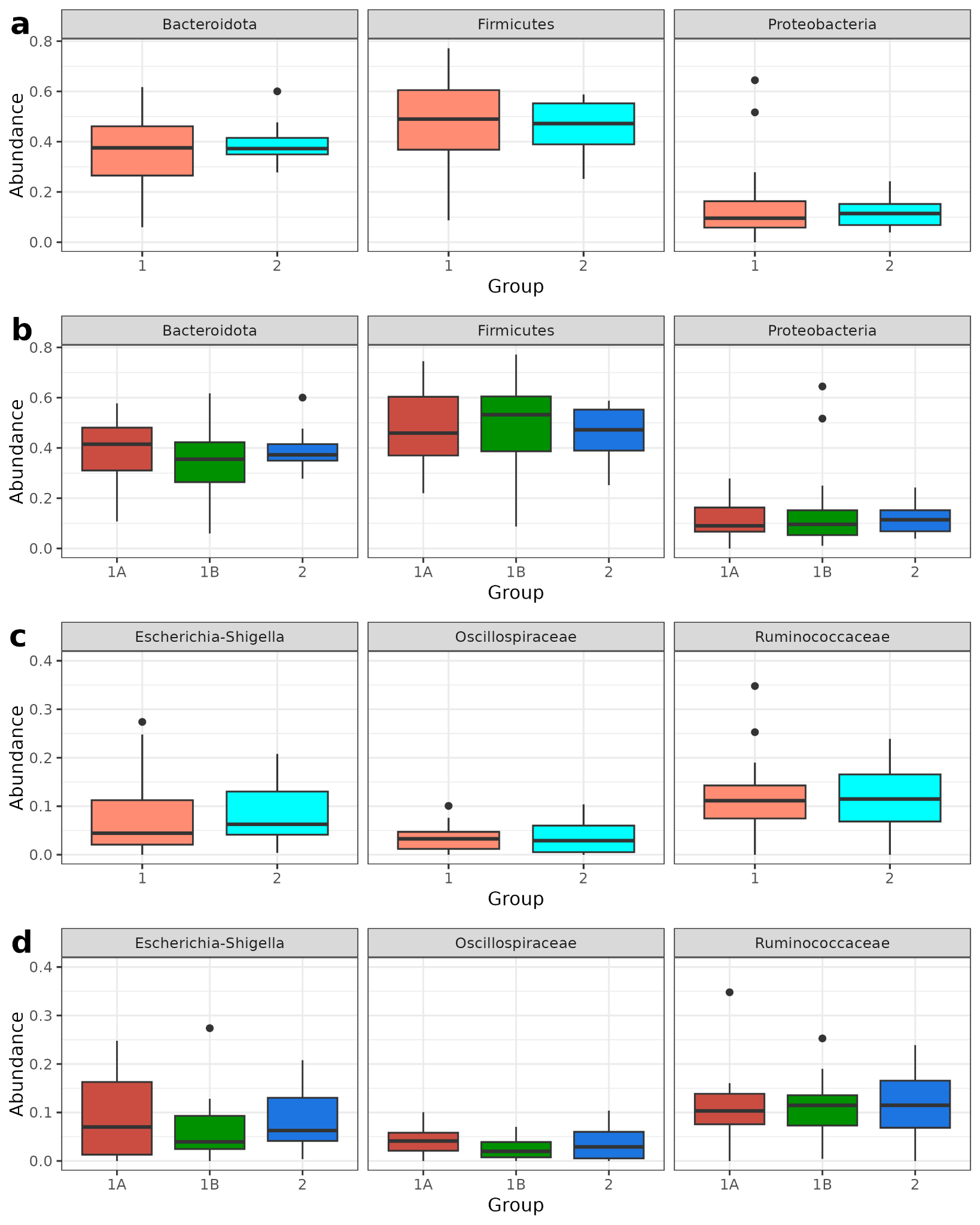


**Figure S3. Relative abundance of bacterial phylum and its relation with the clinical classification of TD.** a) Relative abundance of the three main bacterial phyla divided divided into a) two groups (TD and no TD; Group 1 and Group 2); and b) when participants are divided into three groups (Confirmed TD, Probable TD and no TD; Group 1A, Group 1B and Group 2, respectively). Panels c) and d) show the abundance of those bacterial families and genus that were statistically associated with LCN2 levels, for participants divided into two groups (c) and participants divided into three groups (d), as commented above.
